# Circulating Tumor DNA as a Cancer Biomarker: An Overview of Biological Features and Factors That may Impact on ctDNA Analysis

**DOI:** 10.3389/fonc.2022.943253

**Published:** 2022-07-20

**Authors:** Estela Sánchez-Herrero, Roberto Serna-Blasco, Lucia Robado de Lope, Víctor González-Rumayor, Atocha Romero, Mariano Provencio

**Affiliations:** ^1^ Liquid Biopsy Laboratory. Biomedical Sciences Research Institute Puerta de Hierro-Majadahonda, Majadahonda, Spain; ^2^ +D Department, Atrys Health, Barcelona, Spain; ^3^ Medical Oncology Department, Hospital Universitario Puerta de Hierro-Majadahonda, Majadahonda, Spain

**Keywords:** ctDNA= circulating tumor DNA, ctDNA kinetics, biomarker, liquid biopsy, monitoring

## Abstract

Cancer cells release nucleic acids, freely or associated with other structures such as vesicles into body fluids, including blood. Among these nucleic acids, circulating tumor DNA (ctDNA) has emerged as a minimally invasive biomarker for tumor molecular profiling. However, certain biological characteristics of ctDNA are still unknown. Here, we provide an overview of the current knowledge about ctDNA biological features, including size and structure as well as the mechanisms of ctDNA shedding and clearance, and the physio-pathological factors that determine ctDNA levels. A better understanding of ctDNA biology is essential for the development of new methods that enable the analysis of ctDNA.

## Introduction

Cancer ranks as the leading cause of death worldwide and the main barrier that hinders life expectancy (1). The emergence of precision medicine in the field of medical oncology brought a halo of hope for cancer patients and has improved notably in the past few decades due to the rapid expansion of knowledge in cancer genomics and the identification of targetable genomic biomarkers (2). Although the discovery of therapeutic biomarkers marked a turning point in cancer patients’ treatment, several challenges arose with them. For example, in lung cancer patients, the increasing number of biomarkers to be assessed compromises the availability of tumor tissue. Moreover, tissue biopsy, apart from being a very invasive procedure that can imply potential complications for the patients, does not reflect tumor heterogeneity, making it more difficult to have an overview of the molecular characteristics of the tumor (3, 4).

In this scenario, liquid biopsy arose as a minimally invasive approach, particularly useful when tumor tissue is inadequate or non-existent, that enables the identification of significant tumor-derived biomarkers throughout the course of the disease, including resistance mutations (5, 6). Different components can be isolated from body fluids and used in liquid biopsy analysis such as circulating tumor cells (CTCs), extracellular vesicles (EVs), tumor-educated platelets (TEPs), or circulating tumor DNA (ctDNA) (7). Among them, we are going to focus on ctDNA as it is the biomarker with more diagnostic and prognostic potential.

**Figure 1:**
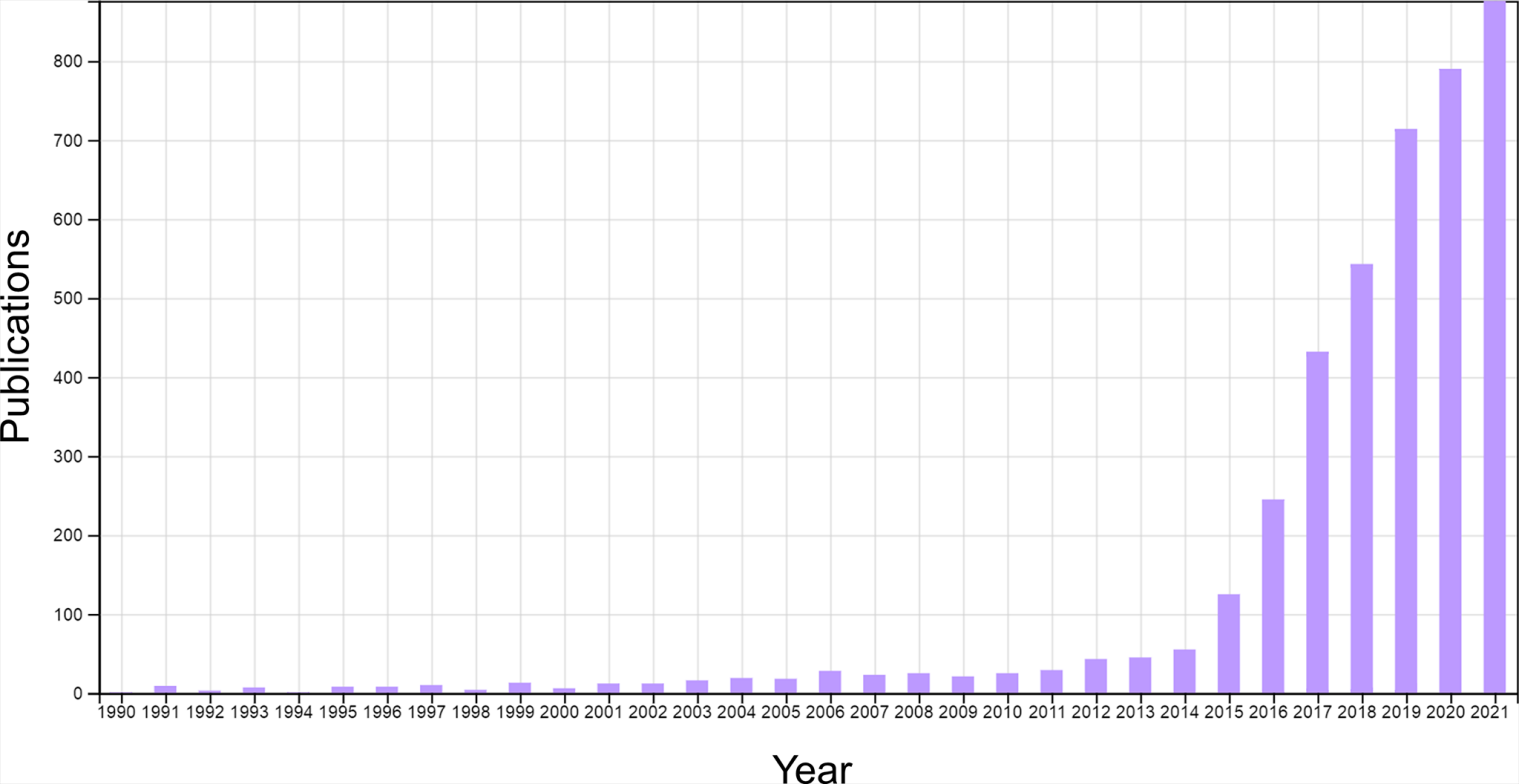
ctDNA publications: Number of publications with ctDNA as a topic between 1990 and 2021, collected in Web of ScienceTM database.

The cell free-DNA (cfDNA) was first described in healthy individuals by Mandel et al. in 1948 (8) and it was not until the year 1977 that Leon et al. found out increased levels of cfDNA in the serum of cancer patients (9), highlighting its huge potential as a tumor biomarker. cfDNA is generally at a concentration between 0 and 100 ng/mL in the blood of healthy patients, and is upped to >1000 ng/mL in cancer patients (4, 10). The fraction of plasma cfDNA derived from tumor cells, known as ctDNA, is the most extensively studied and the most used non-invasive alternative, from a clinical point of view, for the molecular characterization of solid tumors, including non-small-cell lung cancer (NSCLC), colorectal cancer (CRC), breast cancer (11), head and neck (12) and melanoma (13). ctDNA was first validated in clinical oncology by examining the *KRAS/BRAF* mutation in CRC patients (14) and then, it was introduced into clinical practice for the detection of mutations in the *EGFR* gene in NSCLC (15). Since then, the interest in this biomarker has exponentially risen being the topic of more than 870 publications in 2021 (Web of Science™ database, **Figure 1**) and being currently used in 359 different trials, listed in the database ClinicalTrials.gov.

Although liquid biopsies often refer to blood biopsies, other biofluids such as urine, saliva, cerebrospinal fluid (CSF), pleural effusion, pericardial effusion, and ascites effusion, can be also used (16, 17). In this way, malignant effusions that occur as a consequence of disease progression are highly informative. Indeed, tumors shed higher amounts of ctDNA into nearby body fluids than into the bloodstream (18). Moreover, peritoneal washings, which are routinely performed in surgeries of ovarian cancer patients, have been shown to be useful for BRCA testing (19). Therefore, although obtaining these biofluids may be a more aggressive procedure, they constitute an informative source for biomarker testing (18).

The study of ctDNA has multiple potential uses in oncology such as early diagnosis, tumor molecular profiling, or early detection of resistance mutations. ctDNA levels correlate well with tumor bulk and therefore it can be used as a surrogate for tumor size and staging (20, 21). In the same way, fluctuations in ctDNA levels have been shown to correlate well with the course of the disease, being an adequate approach for noninvasive tumor response to treatment monitoring for many cancer types (22–24).

However, the structure and origin of ctDNA, as well as the mechanisms of ctDNA shedding, filtering, degradation, and clearance remain unclear. In this review, we summarize the dynamics of extracellular tumor DNA, including the balance between ctDNA release and clearance and the influence of clinicopathological factors in these processes.

## ctDNA: Characteristics and Mechanisms of Release

cfDNA comprises small fragments of double-stranded nuclear (coding and non-coding) and mitochondrial DNA (mtDNA) of approximately 40-200 base pairs (bp) in size, with a peak at about 166 bp that corresponds with nucleosome-associated DNA fragments (4, 25). Although the main source of cfDNA is the hematopoietic system (55% white blood cells and 30% erythrocyte progenitors) (25), there is still a huge interest in understanding how different organs contribute to the overall amount of cfDNA in the physiological and pathological conditions.

**Figure 2:**
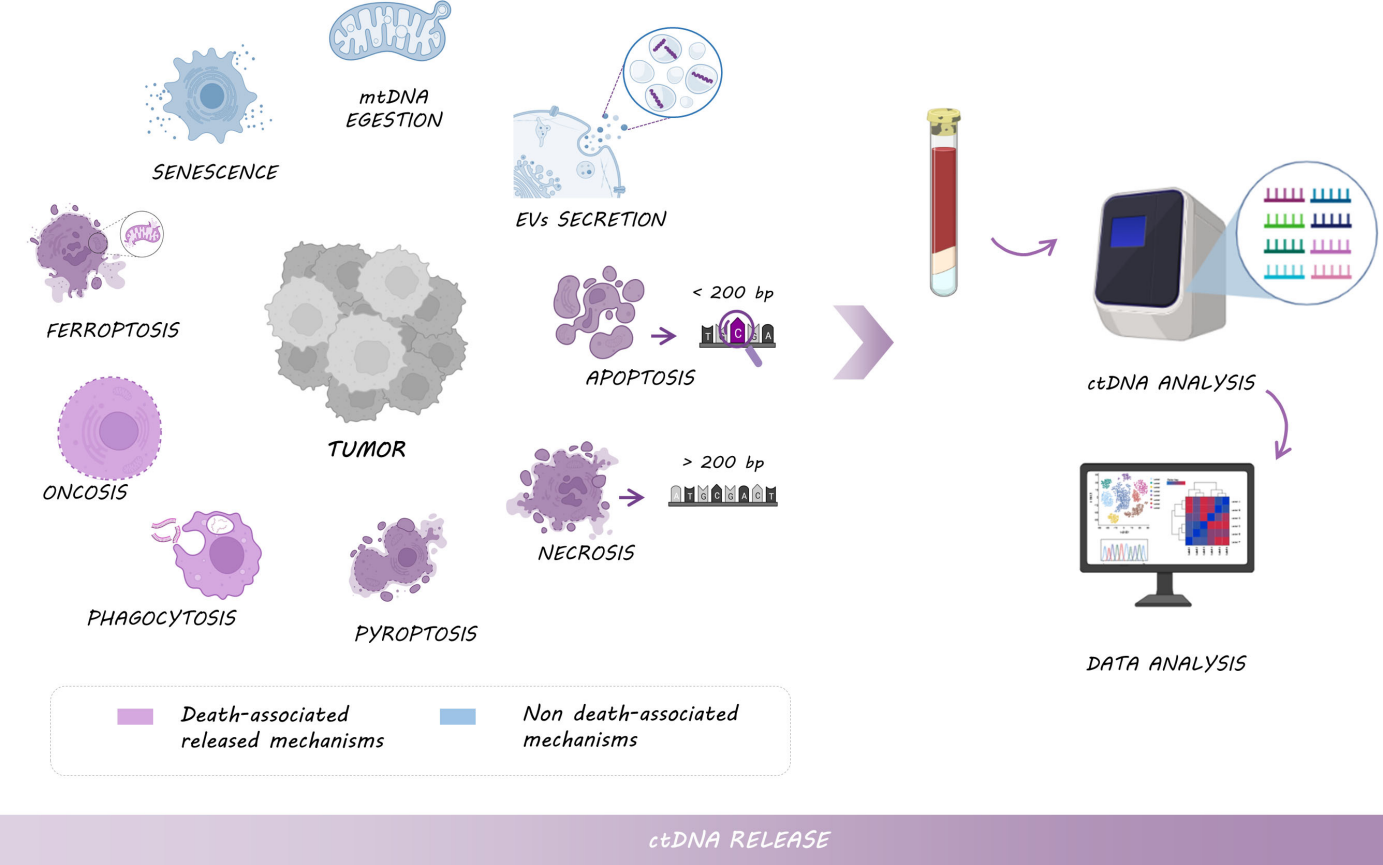
Mechanisms of ctDNA release: Tumor cells shed DNA into the bloodstream by different types of cell death, including necrosis (larger fragments of >200 bp), apoptosis (shorter fragments of <200 bp enriched with tumor-derived genomic alterations), pyroptosis, phagocytosis, oncosis or ferroptosis, but also by non-death associated mechanisms such as senescence, or the active secretion of EVs and mtDNA. Thus, ctDNA analysis provides tumor-relevant clinical information.

ctDNA can be released by a multitude of mechanisms, not only when cells die *via* apoptosis, necrosis, oncosis, ferroptosis, pyroptosis, and phagocytosis, but also by senescence or the active secretion in extracellular vesicles (EVs) and mtDNA egestion (26, 27) (**Figure 2**).

Fragment length and nucleosome occupancy might provide clues for cfDNA shedding mechanisms. Indeed, short fragments of <200 bp are assumed to be released during apoptosis as a consequence of caspase-dependent cleavage. Multiple of these fragments are packed in apoptotic blebs and phagocytized by macrophages, to be finally released into the blood and lymphatic circulation (28, 29). Interestingly, shorter fragments (<100 bp) might be enriched with ctDNA and mtDNA which preferentially carried tumor-derived genomic alterations (30, 31). Indeed, a higher ctDNA fragmentation pattern was observed in melanoma, lung cancer, and metastatic CRC patients with high levels of mutation burden compared with healthy individuals (32). Conversely, large fragments of >200 bp are originated during the necrosis process (33), however, the contribution of necrosis in the amount of cfDNA remains unclear (34). Interestingly, DNA of necrotic cells can be further degraded by DNase I, and necrotic cells can be engulfed by macrophages, originating smaller fragments of circulating DNA (35, 36). For all those reasons, the development of methods based on cfDNA size and fragmentation pattern is crucial to enhance the enrichment of ctDNA and consequently, improve the sensitivity of methods for ctDNA analysis. Furthermore, the distribution of cfDNA fragments with different sizes is important, since it reflects cfDNA integrity. Although ctDNA derived from apoptotic bodies would be more informative in terms of tumor molecular information, cfDNA integrity seems to be higher in cancer patients compared to healthy individuals, suggesting that necrotic cell death plays an important role in ctDNA release, especially in advanced stages and aggressive tumors (37, 38). This observation could be explained by the fact that healthy cells die primarily by apoptosis, while malignant cells die not only from apoptosis but also from necrosis or autophagy (9). In this regard, cancer cells could activate autophagy to obtain an alternative energy source from the digestion of their damaged organelles or their self-digestion, shedding ctDNA as a consequence (39).

DNA from necrotic or apoptotic cells can be also released into the circulation by different immune cell types; however, it is not clear how much each mechanism contributes to the amount of ctDNA. After the phagocytosis of necrotic and apoptotic cells, macrophages or other scavenger cells digest the DNA into smaller fragment sizes and release them into the tissue microenvironment and bloodstream, actively or dying (36, 40).

ctDNA can also be actively released by living tumor cells, from primary tumors or metastases *via* EVs. Like cfDNA, there is a wide variety of EVs in terms of size whose role in cancer and, specifically, in the transport of ctDNA between distant tissues for cell communication seems to differ. In this sense, Vagner et al. showed that both ctDNA and EVs size seems to be a key element in genomic alteration transport (41). Indeed, large vesicles (from 100 nm up to 1 μm in diameter) from prostate cancer patients, such as microvesicles or apoptotic bodies, appear to be enriched with smaller fragments of ctDNA (<200 pb), compared with small EVs, from 30 to 150 nm in diameter, such as exosomes (41, 42). Still, nanoscale EV-derived DNA (approximately 114 nm average size in stage-I EV samples) has been demonstrated to be a superior mutation detection method in early-stage NSCLC compared to cfDNA (43). In line with these data, additional studies have identified the presence of DNA in EVs isolated from cancer patient samples and described the identification of different mutations in oncogenes such as *KRAS* or *TP53* (44–47). However, the proportion of ctDNA engulfed into EVs actively released by tumor cells and the effect of different treatments on this active secretion is not clear (41, 48).

**Figure 3:**
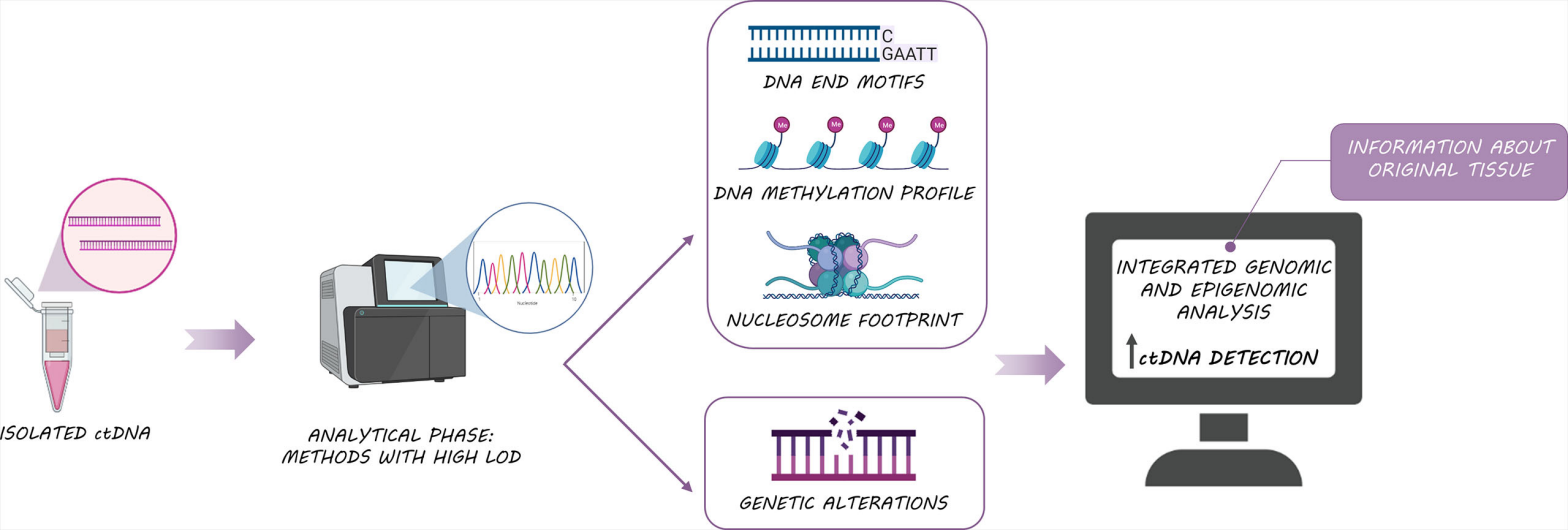
Biological features of ctDNA: The integrated analysis of ctDNA somatic alterations, methylation and fragmentomic information, improves ctDNA detection and provides useful information about original tissue.

Irrespective of the mechanism of ctDNA shedding; nucleosome footprints, DNA methylation profiles, DNA preferred end motifs, and genetic alterations can be used to characterize and identify the origin of cfDNA as they carry information from the original tissues (49–51) (**Figure 3**). In this regard, certain human genomic locations have been described as preferential ends when ctDNA is generated (52), suggesting that DNA cleavage is a non-random process. Interestingly, a greater end motif diversity has been associated with cancer patients (53, 54), suggesting that ctDNA tail motifs could be used to enhance the performance of cancer diagnosis by identifying the fragments of cfDNA from tumor cells (ctDNA) and filtering out fragments from healthy cells. Another interesting approach for determining the tissue source of ctDNA was proposed by Snyder et al. (49), who hypothesized that it is feasible to identify cfDNA origin based on nucleosome positioning. Nucleosomes are distributed along DNA following different patterns that correlate with characteristic epigenetic features of different cell types or even according to cancer types. Matching the epigenetic footprint of these ctDNA fragments against reference databases would enable the molecular classification of cancers of unknown origin. In line with these data, the stability of DNA methylation and the presence of cell-specific methylation patterns can also contribute to the identification of tumor origin or even the detection of metastasis through cfDNA analysis (55). Specifically, the analysis of differentially methylated regions in colon and liver tissues enabled the differentiation of patients with liver or colon cancer but also, the discrimination between colon cancer patients with and without liver metastasis (56). Indeed, the analysis of cfDNA methylation has been already approved by the FDA for its use in the clinic (57), being the Epi proColon test the first screening analyzing a cfDNA methylation biomarker approved in 2016 for colorectal cancer patients (58).

## ctDNA Clearance

**Figure 4:**
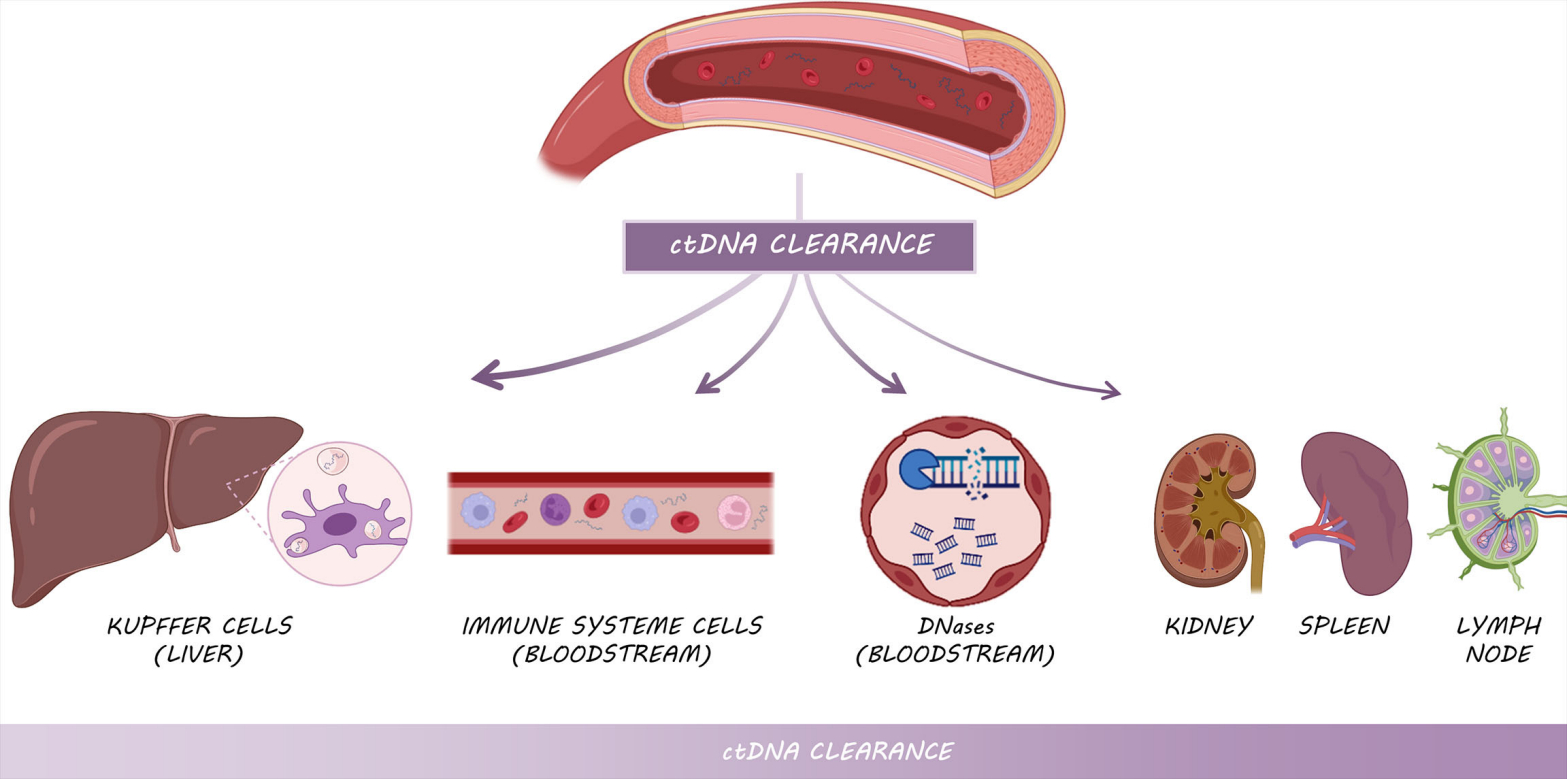
Mechanisms of ctDNA clearance: Kupffer cells from the liver are primarily responsible for ctDNA clearance, followed by circulating enzymes and immune system cells and other filtering organs such as kidneys, spleen and lymph nodes.

The amount of cfDNA, and ctDNA in particular, depends on a balance between DNA shedding and DNA clearance. Overall, the half-life of cfDNA ranges from 16 minutes to 2.5 hours (59), as a consequence of the action of three main different mechanisms: (i) the action of DNases present in the bloodstream (60), (ii) the active clearance of nucleosomes and DNA and (iii) filtration in organs such as kidney or lymph nodes (**Figure 4**).

ctDNA clearance can be carried out by various filtering organs (60). Kupffer cells within the liver are responsible for clearing the majority of cfDNA, specifically longer fragments (61, 62), followed by kidneys, which are involved in DNA fragmentation through their deoxyribonuclease activity (62). In this way, *in vivo* experiments injecting radiolabeled mononucleosomes in mice demonstrated that the liver removed approximately 70 to 85% of the nucleosomes within 10 min (61). The macrophages of the spleen and lymph nodes play also a minor role in ctDNA clearance (**Figure 4**). In addition to these organs, lymphatic drainage may constitute the main source of ctDNA clearance within the tumor microenvironment (60). In cancer patients, cfDNA level is higher than in healthy individuals in part due to the excess of cell death by the whole set of mechanisms aforementioned, which leads to the overload of the clearance systems and subsequent accumulation. Nevertheless, the kinetic dynamics of ctDNA in cancer patients need to be further studied.

Finally, the association with molecular or macromolecular complexes, as well as encapsulation in EVs, prevent the rapid degradation of ctDNA by circulating enzymes and immune system cells (63). Another factor that seems to play a role in ctDNA clearance is fragment size, but it is still not clear how they affect half-life.

## ctDNA Levels in Different Types of Cancer

**Figure 5:**
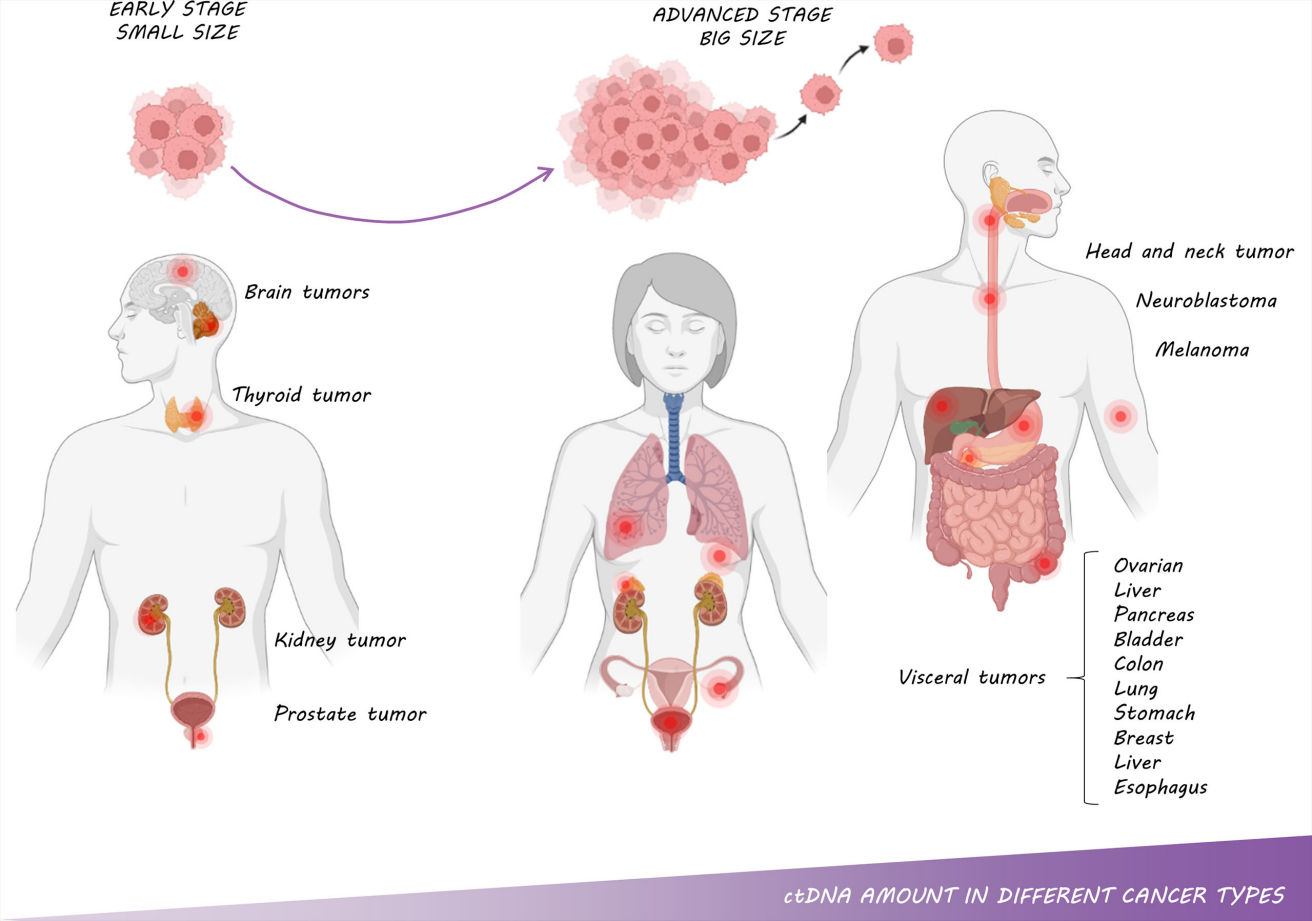
ctDNA amount in different cancer types: The amount of ctDNA is correlated with tumor type, size, stage and metastasis.

The first time that cfDNA was measured in different cancer types was in 1977 by Leon et al. (9), who reported that levels of cfDNA in patients with various cancers were higher compared with healthy individuals. A few years later, in 1989, Stroun et al. (64) stated that increased levels of cfDNA in cancer patients were caused by a fraction of DNA released into the bloodstream by cancer cells, this portion of cfDNA was named ctDNA. Nowadays, it is well established that ctDNA levels vary depending on the cancer type (**Figure 5**). It has been especially characterized that tumors located in the central nervous system release the lowest levels of ctDNA into the bloodstream due to the blood-brain barrier (21, 65, 66). Of note, more than 90% of patients with gliomas did not harbor detectable levels of ctDNA according to Huang et al. (67). Similarly, Zill et al. (68) analyzed 25,578 samples from 21,807 patients in more than 50 tumor types, reporting a ctDNA detection rate of 93%. Remarkably, no differences were found in terms of ctDNA detection except for patients with brain tumors or brain-only metastases, who shed significantly less ctDNA into the bloodstream. Likewise, some studies have also pointed out that patients with visceral metastases have higher levels of ctDNA than those with brain metastases (69–72). Noteworthy, about 30% of cancer patients develop intracranial metastases, a severe complication that decisively affects the patient’s prognosis and quality of life (73,74). Thus, it would be important to optimize the detection of ctDNA for these patients in other body fluids such as CSF. In addition, ctDNA detection is rather challenging in medulloblastomas, or kidney, prostate or thyroid cancer. On the other hand, ctDNA can be easily detected in samples from advanced stages of ovarian, liver, pancreas, bladder, colon, lung, stomach, breast, liver, esophagus, and head and neck cancer patients as well as neuroblastoma and melanoma patients (21).

Interestingly, there are also variations within the same type of cancer depending on tumor histology. For example, in lung cancer, a higher percentage of ctDNA detection has been described in squamous tumors compared to adenocarcinomas. The most plausible explanation is that squamous tumors have a more necrotic profile (75). These results have also been observed in patients with triple-negative breast cancer, whose ctDNA levels are higher than those of other breast cancer subtypes, which can be related to a higher rate of necrosis and cell proliferation (76, 77).

Lastly, tumors harboring certain alterations such as *TP53* mutations and copy number gains seem to have increased ctDNA shedding, which may be due to increased metabolic activity or cellular turnover (71). In this regard, *TP53* alterations have been suggested to be markers of aggressiveness and poor prognosis (78).

## ctDNA as a Surrogate of Tumor Burden, Stage, and Metastasis

The amount of ctDNA has been associated with tumor size, stage, and metastasis in multiple studies (**Figure 5**). Specifically, a retrospective study of serially collected liquid biopsy samples from 40 ovarian cancer patients demonstrated a significant correlation between lesion volume and ctDNA level (79). Another study that analyzed samples from 640 patients with different tumor types described a clear correlation between ctDNA and cancer stage, reaching higher levels in patients with advanced disease and lower levels in premalignant and early-stage cancers (21). In line with these results, analyzing samples from more than 20,000 patients with different tumors, Zill and colleagues showed that those patients with premalignant lesions or earlier stages shed less ctDNA than those with advanced stages (68). Specifically, in NSCLC patients, Chabon et al. were able to detect ctDNA in 42%, 67%, and 88% of patients with stage I, II, and III diseases respectively (80). In fact, 50% of localized tumors shed ctDNA without reaching 0.01% of the ctDNA level (80); whereas advanced-stage tumors release concentrations of ctDNA than can exceed 10% of the cfDNA (21).

On the other hand, in ctDNA-positive patients, tumor size and volume correlate broadly with ctDNA levels, as measured by the mean of variant allele frequency (VAF) of single nucleotide variants detected in plasma ctDNA (81). Currently, it is not well established how ctDNA should be quantified. This issue is especially controversial in tumors that do not harbor druggable mutations. In this sense, it is not clear whether it is more appropriate to select the highest VAF among all detected mutations or to take all of VAFs into account through summation, arithmetic mean, or other approaches.

In summary, ctDNA levels increase proportionally according to tumor burden, disease stage, and metastasis, highlighting the use of ctDNA as a prognostic biomarker. Indeed, it is well established that patients with high levels of ctDNA have worsened survival outcomes compared with those with lower or even undetectable levels of ctDNA (82–85).

## ctDNA to Monitor Treatment Outcomes

Numerous studies show that ctDNA levels correlate well with tumor load and therefore ctDNA dynamics can be used as a surrogate of treatment response (86–88). In addition, the modification of the ctDNA methylation profile has been proposed as an alternative biomarker for treatment response (89). It has been shown that the type of treatment, as well as the time interval between exposures and the dose, may rate affect ctDNA shedding. In this way, it has been suggested that targeted therapies used in cancer patients, such as *EGFR* or *ALK* tyrosine kinase inhibitors, promote faster ctDNA clearance than immunotherapy (90). Furthermore, cytotoxic therapies such as chemotherapy or ionizing radiation seem to increase cfDNA levels due to cellular senescence (91, 92). Of note, it is well established that some chemotherapy agents produce leukopenia. cfDNA from dying cells dilutes ctDNA in wild-type (wt) DNA leading to decreased levels in VAF, which may bias results. Conversely, other cancer treatments do not release as much cfDNA due to its mainly cytostatic effect, implying cell growth arrest (93). In the neoadjuvant setting, ctDNA has been shown to correlate well with tumor response to treatment. Recently, NADIM investigators have shown that ctDNA clearance after neoadjuvant chemo-immunotherapy outperformed tumor response to treatment measured by CT-scans and according to RECIST criteria in the prediction of survival (94). Similarly, a significant association between pathological complete response and ctDNA clearance was reported in the CheckMate 816 trial (95). Measurement of residual disease following neoadjuvant treatment that accurately predicts long-term survival is an essential requirement for clinical trial development. Although further studies are needed, ctDNA postulates as an early surrogate of survival being a promising trial endpoint in the neoadjuvant setting.

Finally, patients with surgically resected tumors show a sharp drop in ctDNA levels after surgery (59). However, the amount of nonspecific cfDNA increased after tumor resection (96), due to injury of surrounding tissue during surgery. In this sense, the appropriate time point for plasma collection after surgery needs to be established. In these patients, ctDNA detection allows monitoring of minimal residual disease (MRD) after tumor resection (97). Several platforms with exceptional sensitivities such as cancer personalized profiling by deep sequencing (CAPP-Seq) (98) targeted error correction sequencing (TEC-Seq) (99), the Tracking Cancer Evolution Through Therapy (TRACERx, Signatera) (81, 100) or CancerSEEK5 multiplex PCR (mPCR) (101) have been shown to be useful for detection of minimal residual disease (MRD) or early detection of cancer. In this regard, it appears that measuring not just ctDNA can boost sensitivity. Combining ctDNA analysis with the study of informative methylation regions improves sensitivity (102) (**Figure 3**).

## Technical Factors Affecting ctDNA Detection

The use of ctDNA to noninvasively assess tumor genomic variants is increasing. However, some pre-analytical and analytical issues may affect the detection and quantification of ctDNA.

Regarding starting material, blood plasma is a preferential choice compared with serum because wt cfDNA released from leukocytes during the clotting process in serum samples dilutes ctDNA in wt DNA (103, 104). Particularly, Soo et al. reported a higher level of cfDNA in serum (481 ng/mL) than in plasma (17.7 ng/mL). Of note in a cohort of 33 pre-treatment serum and 75 pre-treatment plasma samples from patients with diffuse large B cell lymphoma, Soo et al. were able to detect more genomic alterations in plasma samples (186 *vs*. 22 mutations) with higher tumor allele fraction (2.8% *vs*. 0.85%) (105), compared with serum samples. In addition, plasma samples have shown less inter-patient variability (106). The use of K_2_ EDTA tubes to collect plasma samples is therefore recommended when samples are processed within 6 hours after blood extraction (107). For longer periods between extraction and processing, the use of special collection tubes with stabilizing agents is recommended. Of note successful preservation of cfDNA over 14 days at room temperature is possible using collection tubes with stabilizing agents (108).

Concerning sample processing, the complete removal of any cellular component is essential. For this goal, the best option is a two-step centrifugation at 1600g for 10 minutes for plasma isolation (109). According to this recommendation, Herrera et al. reported less concentration of cfDNA in plasma samples that were centrifuged twice compared with samples that were centrifuged only once (13 µg/l *vs.* 819 µg/l), revealing that cfDNA concentrations were contaminated with genomic DNA (110). These observations confirm that the second centrifugation step is crucial for ctDNA analysis. Finally, it is well known that ctDNA integrity is better conserved as cfDNA extracts compared to plasma when samples are stored at -80°C and avoiding freeze-thaw cycles (103).

As already mentioned, body fluids other than blood have shown a higher concentration of cfDNA compared to blood samples in patients with lung adenocarcinoma with *EGFR* mutations (1.90 vs. 0.36 ng/µL; p=0.0130). Likewise, CSF from patients with primary brain tumors such as glioblastoma, glioma, or primary central nervous system lymphoma showed higher amounts of ctDNA compared to peripheral blood (18, 19).

Technical procedures for cfDNA isolation can be classified into three categories: phase isolation, silicon membrane-based spin column, and magnetic bead-based isolation. Phase isolation methods may lead to a high cfDNA isolation yield and a wide range of DNA fragment sizes. On the other hand, spin column and magnetic bead-based methods have lower efficiency but show a higher selective recovery for DNA fragments of a certain size (111, 112). Specifically, cfDNA purification using magnetic beads appears to recover higher amounts of small cfDNA fragments compared to silica membrane methods (113). In any case, automated processing should be performed to reduce operator variability (114). Nevertheless, within automatic methods, Pérez-Barrios et al. reported different recovery of mono-, di- and tri-nucleosomes DNA fragments when analyzing 34 cfDNA samples obtained from 17 plasma samples from cancer patients extracted by Maxwell^®^ RSC ccfDNA Plasma Kit (Promega Corporation, Madison, WI, USA) and MagNA Pure Compact Nucleic Acid Isolation Kit I (Roche Diagnostics, Penzberg, Germany) methodologies (112).

Currently, several platforms are available for noninvasive biomarker testing some of which have received approval from regulatory agencies. There is a wide range of reported sensitivities of the different methodological approaches, in this way PCR-based approaches have a significantly lower limit of detection (LOD) compared to other technologies such as dPCR and NGS (115). Although dPCR offers an ultra-high sensitivity for ctDNA analysis, only a few known mutations can be tested at a time, whereas NGS technologies allow the screening of multiple genomic alterations, known or unknown. In addition, NGS enables the combination of genomic data and epigenomic signatures, which may improve sensitivity (116) (**Figure 3**). In any case, the knowledge of the limitations of the different technical approaches for ctDNA analysis is crucial for the accurate interpretation of the results (117).

cfDNA input remains the major limiting factor, and for most techniques using less than 20 ng of cfDNA may impair results. A study by Zhang Y et al. showed that the sensitivity declined from 82.6% to 46.7% when using cfDNA inputs of ≥ 5 ng per reaction and < 2 ng, respectively (118). Furthermore, several comparative studies have clearly reported that, among other technical factors, discordant calls mostly occur at low VAF (115, 119), and therefore VAFs should always be reported in clinical reports.

Finally, it is important to point out that clonal hematopoiesis (CH) constitutes an important source of false-positive calls. CH is defined by the presence of a somatic mutation in blood or hematopoietic progenitor cells, but without other diagnostic criteria for hematological malignancy. It is more frequent in aged patients and patients with solid tumors and of course, it is more likely to be detected with deeper sequencing approaches (120). Importantly, CH-derived mutations can lead to erroneous sequencing results which thereby might guide erratic treatment recommendations (121).

## Conclusions

Liquid biopsy overcomes some tissue biopsy limitations such as tumor heterogeneity, tissue availability, and risks associated with the invasive procedure. Among the biological components of body fluids, ctDNA has emerged as a pivotal analyte for the management of cancer patients. However, ctDNA detection and quantification are affected by several physio-pathological conditions and a deeper knowledge of factors affecting ctDNA kinetics is needed. The size fragment pattern, nucleosome, and methylation profile of ctDNA may differ according to the original tissue and the mechanism of release, which may be clinically informative, and methodological approaches capable to explode this information are of particular interest.

## Author Contributions

ES-H, RS-B and AR drafted and critically revised the manuscript. All authors reviewed the draft. All authors contributed to the article and approved the submitted version.

## Funding

ES-H was funded by the Consejería de Ciencia, Universidades e Innovación of the Comunidad de Madrid (Doctorados Industriales of the Comunidad de Madrid IND2019/BMD-17258). RS-B was funded by the Ministerio de Ciencia e Innovación (Programa Estatal de Investigación, Desarrollo e Innovacion Orientada a los Retos de la Sociedad, RTC2019-007359-1).

## Conflict of Interest

Authors ES-H and VG-R were employed by Atrys Health. AR reports the following conflict of interest: Consulting or Advisory Role: Takeda, AstraZeneca. Research Funding: Bristol Myers Squibb Foundation (Inst), Boehringer Ingelheim (Inst), Takeda (Inst) Expert Testimony: Vivo Diagnostics. MP reports grants, personal fees, and travel expenses from BristolMyers Squibb, Roche, and AstraZeneca; and personal fees from Merck Sharpe & Dohme and Takeda, outside the submitted work.

The remaining authors declare that the research was conducted in the absence of any commercial or financial relationships that could be construed as a potential conflict of interest.

## Publisher’s Note

All claims expressed in this article are solely those of the authors and do not necessarily represent those of their affiliated organizations, or those of the publisher, the editors and the reviewers. Any product that may be evaluated in this article, or claim that may be made by its manufacturer, is not guaranteed or endorsed by the publisher.

## Abbreviations

bp, base pair; cfDNA, cell-free DNA; CH, clonal hematopoiesis; CRC, colorectal cancer; CTC, circulating tumor cell; CSF, cerebrospinal fluid; ctDNA, circulating tumor DNA; ddPCR, digital droplet PCR; dPCR, digital PCR; EVs, extracellular vesicles; LOD, limit of detection; MRD, minimal residual disease; mtDNA, mitochondrial DNA; NGS, next-generation sequencing; NSCLC, non-small-cell lung cancer; VAF, variant allele frequency; wt, wild-type; *ALK*, ALK receptor tyrosine kinase; *BRAF*, B-Raf proto-oncogene, serine/threonine kinase; *EGFR*, epidermal growth factor receptor; *KRAS*, KRAS proto-oncogene, GTPase; *TP53*, tumor protein p53.
